# COVID-19 and possible links with Parkinson’s disease and parkinsonism: from bench to bedside

**DOI:** 10.1038/s41531-020-00123-0

**Published:** 2020-08-20

**Authors:** David Sulzer, Angelo Antonini, Valentina Leta, Anna Nordvig, Richard J. Smeyne, James E. Goldman, Osama Al-Dalahmah, Luigi Zecca, Alessandro Sette, Luigi Bubacco, Olimpia Meucci, Elena Moro, Ashley S. Harms, Yaqian Xu, Stanley Fahn, K. Ray Chaudhuri

**Affiliations:** 1 Departments of Psychiatry, Neurology, Pharmacology, Columbia University Medical Center, New York State Psychiatric Institute, New York, NY 10032 USA; 2 Department of Neuroscience, Parkinson and Movement Disorders Unit, University of Padua, Padua, Italy; 3 King’s College London, Institute of Psychiatry, Psychology & Neuroscience, De Crespigny Park, London, SE5 8AF UK; 4 Parkinson’s Foundation Centre of Excellence, King’s College Hospital, Denmark Hill, London, SE5 9RS UK; 5 Department of Neurology, Vagelos College of Physicians and Surgeons, Columbia University and the New York Presbyterian Hospital, New York, NY 10032 USA; 6 Department of Neurosciences, Thomas Jefferson University, Philadelphia, PA 19107 USA; 7 Department of Pathology and Cell Biology, Vagelos College of Physicians and Surgeons, Columbia University and the New York Presbyterian Hospital, New York, NY 10032 USA; 8 Institute of Biomedical Technologies, National Research Council of Italy, Segrate, Milan, Italy; 9 Division of Vaccine Discovery, La Jolla Institute for Allergy and Immunology, La Jolla, CA 92093 USA; 10 Department of Medicine, University of California, San Diego, CA 92093 USA; 11 Department of Biology, University of Padova, Padova, Italy; 12 Department of Pharmacology and Physiology, Drexel University College of Medicine, Philadelphia, PA 19102 USA; 13 Center of Neuroimmunology and CNS Therapeutics, Institute of Molecular Medicine and Infectious Diseases, Drexel University College of Medicine, Philadelphia, PA 19102 USA; 14 Department of Microbiology and Immunology, Drexel University College of Medicine, Philadelphia, PA 19102 USA; 15 Department of Neurology, Grenoble Alpes University Hospital, Grenoble, France; 16 Grenoble Institute of Neurosciences GIN-INSERM U1216/CEA/UGA, Grenoble, France; 17 Grenoble Alpes University, Grenoble, France; 18 Department of Neurology, Center for Neurodegeneration and Experimental Therapeutics, University of Alabama at Birmingham, Birmingham, AL 35294 USA; 19 Department of Psychiatry, Columbia University Irving Medical Center, New York, NY 10032 USA

**Keywords:** Translational research, Translational research, Translational research, Translational research

## Abstract

This Viewpoint discusses insights from basic science and clinical perspectives on coronavirus disease 2019 (COVID-19)/severe acute respiratory syndrome-coronavirus-2 (SARS-CoV-2) infection in the brain, with a particular focus on Parkinson’s disease. Major points include that neuropathology studies have not answered the central issue of whether the virus enters central nervous system neurons, astrocytes or microglia, and the brain vascular cell types that express virus have not yet been identified. Currently, there is no clear evidence for human neuronal or astrocyte expression of angiotensin-converting enzyme 2 (ACE2), the major receptor for viral entry, but ACE2 expression may be activated by inflammation, and a comparison of healthy and infected brains is important. In contrast to the 1918 influenza pandemic and avian flu, reports of encephalopathy in COVID-19 have been slow to emerge, and there are so far no documented reports of parkinsonism apart from a single case report. We recommend consensus guidelines for the clinical treatment of Parkinson’s patients with COVID-19. While a role for the virus in causing or exacerbating Parkinson’s disease appears unlikely at this time, aggravation of specific motor and non-motor symptoms has been reported, and it will be important to monitor subjects after recovery, particularly for those with persisting hyposmia.

## Introduction

Over the past twenty years, novel viral epidemics, including influenza, severe acute respiratory syndrome (SARS) and Middle Eastern respiratory syndrome (MERS), have appeared, likely through zoonosis^1–6^. There are few, if any, therapeutic options for treating these disorders and they can induce significant mortality^7,8^. In 2019, a novel coronavirus outbreak, known as COVID-19, was reported in China, and as of May 2020, it had spread to 229 countries^9^.

This coronavirus, known as SARS-CoV-2, is a large enveloped non‐segmented positive‐sense RNA virus^10^. When the SARS-CoV-2 virus, and in particular its Spike (S) protein, makes contact with cells, it binds to a number of host proteins (known as virus receptors) that assist in its entry^10^.

## Symptoms

Like its related family members SARS-CoV and MERS-CoV, SARS-CoV-2 initially presents as a respiratory illness, characterized by cough, dyspnea, fever, and other upper and lower respiratory systems manifestations^11^. However, COVID-19 is associated with a variety of other symptoms and clinical manifestations due to its spread to many other organs and systems^11^.

At this time, it appears that all subjects who have recovered from COVID-19 have developed T cells that recognize specific viral epitopes, including the S protein^12^. The extraordinarily wide range of symptoms and severity, including many infected subjects showing mild or no effects, may be due to cross-reactivity of T cells previously developed in response to prior coronavirus infections that cross-react with SARS-CoV-2, and it is remarkable that nearly half of individuals tested from blood samples prior to 2019 have such cells^12^. It is also possible that different routes of infection, including via the gastrointestinal tract, may result in different symptoms^13^.

Epidemiological and public health studies indicate that infection with the SARS-CoV-2 affects all demographics, but has grave implications for older frail subjects^14^, particularly those with comorbidities as well as Black, Asian, and minority ethnic (BAME) subjects in a disproportionate manner (https://www.england.nhs.uk/coronavirus/workforce/addressing-impact-of-covid-19-on-bame-staff-in-the-nhs/). This is not always the case for viral disorders, as some, like polio, are typically more dangerous for the young^15^. The impression that SARS-CoV-2 infection was particularly pathogenic in older frail subjects has been confirmed by high mortality rates, particularly in residential home patients across the United Kingdom, Italy, the United States, and many other countries^16,17^. Moreover, other comorbidities and factors have been associated with more severe infection, such as diabetes, obesity, pre-existing end organ disease, hypertension, and male sex^18,19^. It has been suggested that the cytokine storm is more easily triggered in patients with chronic inflammation, such as those with diabetes, obesity, and cardiac disease^20^. The cause of high mortality in older BAME subjects reported in the UK and USA remains unclear, although role of comorbidities such as diabetes, hypertension, and obesity as well as social deprivation are implicated (https://www.england.nhs.uk/coronavirus/workforce/addressing-impact-of-covid-19-on-bame-staff-in-the-nhs/).

While the majority of infected people exhibit mild or moderate symptoms and do not require hospitalization, more severe patients need to be hospitalized and sometimes intubated due to severe respiratory distress^21^. Other serious consequences of COVID-19 include acute kidney injury, a coagulopathy similar to disseminated intravascular coagulation^22^, thrombosis^23^, and a newly recognized post-infection syndrome in children, known as multi-system inflammatory syndrome in children potentially associated with COVID-19^24^. The sequelae of each of these syndromes can result in multi-organ failure^11,25^.

A significant number of those diagnosed with COVID-19 have reported a broad spectrum of neurological consequences^26–32^. Neurological symptoms include those associated with dysfunction of the central (fatigue, headache, confusion, stroke^33^, dizziness, syncope^34^, seizure, anorexia, and insomnia)^35–38^, peripheral (anosmia, ageusia, myoclonus^39^, neuropathic pain, and myalgias)^26,35,40^, combined central-peripheral (Guillain Barre syndrome^41^) and enteric nervous systems (diarrhea^13^). Some gastrointestinal manifestations, including diarrhea, may be related to the expression of the viral receptor ACE2 and a serine protease, transmembrane serine protease 2 (TMPRSS2), involved in S protein priming, in the small intestinal epithelia and colon^42^.

**Fig. 1:**
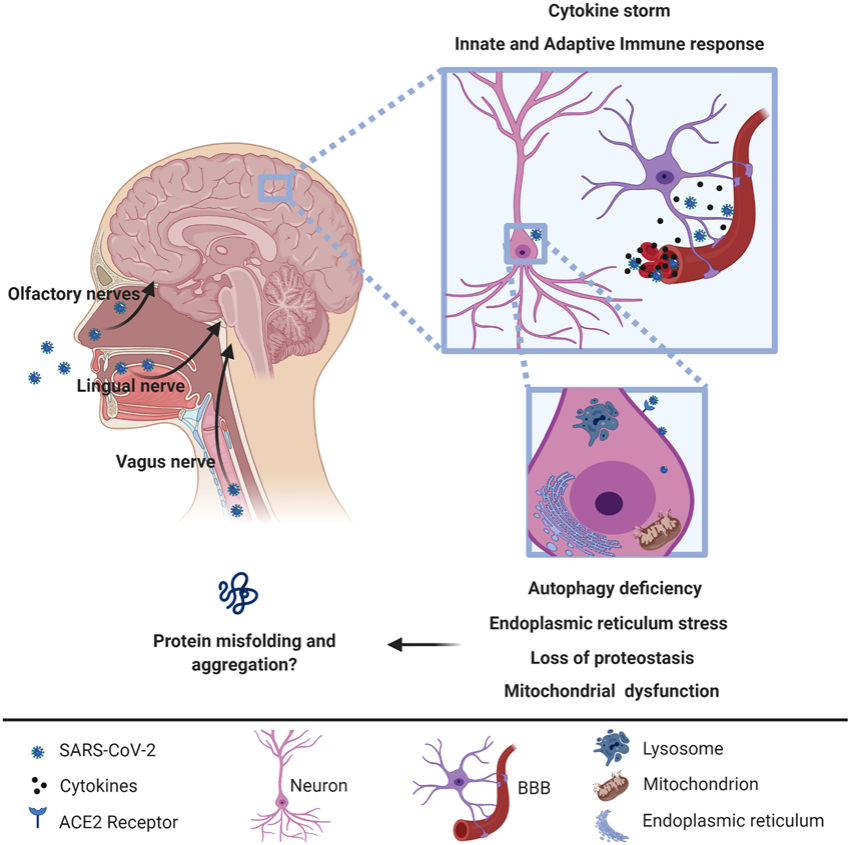
Possible entry routes for SARS-CoV-2 into central nervous system and potential intracellular consequences. There is evidence for SARS-CoV-2 invasion of vasculature in the brain, but little evidence for SARS-CoV-2 in brain parenchyma at this time: this issue will become clearer with results from ongoing autopsy studies. Whether or not the virus is present in neurons or astrocytes, there may be multiple consequences for brain cells, in part through intracellular responses to inflammation that could lead to protein misfolding, a feature of neurodegenerative disorders.

As many as 65% of COVID-19 affected individuals reported hyposmia and ageusia^43^, features that suggest the possibility of trans-synaptic spread via the olfactory, lingual, and glossopharyngeal nerves (Fig. 1), secondary to a respiratory route of infection. Hyposmia is now officially recognized as a symptom of COVID-19 by the UK government and may be a sign in “asymptomatic” carriers who may not have upper respiratory tract symptoms.

A recent review of 43 confirmed COVID-19 cases in a London, UK hospital suggested emergence of specific neurological presentations, including encephalopathies, inflammatory central nervous system syndromes, ischemic strokes, and peripheral neurological disorders, although parkinsonism and rates of hyposmia or ageusia were not reported^44^.

We and others have previously flagged concerns regarding COVID-19 in people with Parkinson’s disease (PD), especially for older and frail subjects with advanced PD who may be particularly vulnerable^45,46^.

## Historical aspects of viruses and parkinsonism

It is remarkable that a relationship between the presence of antibodies to coronaviruses that cause the common cold, coronavirus OC43 and 229E, in the cerebrospinal fluid (CSF) and Parkinson’s disease was reported nearly twenty years prior to the current pandemic by Stanley Fahn and colleagues^47^. Prior coronaviruses have been occasionally reported to exhibit neurological manifestations and CSF invasion^48^, including in children^49,50^.

Medical history provides observations supporting links between viral infections and parkinsonism^51^. The best known example is the post-encephalitic parkinsonism observed during the encephalitic lethargica outbreak that overlapped with the Spanish Flu (influenza A virus H1N1) pandemic in 1918^52^. However, after 100 years, the cause of encephalitis lethargica still remains a mystery^53^. While a causal role of influenza A H1N1 virus in the development of post-encephalitic parkinsonism is not confirmed^52^, an association between influenza A virus infection and development of transient parkinsonism is reported^54^. Notably, the avian flu resulted in parkinsonism in many survivors^55^. Other viral infections have been associated with the development of transient or, more rarely, permanent parkinsonism, including Epstein-Barr, Japanese encephalitis, Coxsackie, West Nile, Western equine encephalomyelitis, and human immunodeficiency virus, mostly due to induction of neuroinflammation and/or hypoxic brain injury with structural/functional damage within the basal ganglia^51,54^ (Table 1). In addition, debated evidence suggests that previous infection with herpes simplex 1, Epstein-Barr, varicella zoster, hepatitis C, and influenza A virus can increase the risk of developing PD in the long-term^54^. Although the “viral hypothesis“ was generally ignored after the discovery of genetic mutations involved in PD pathogenesis, the role of “environmental” factors acting as peripheral triggers of the neurodegenerative process in susceptible individuals has been increasingly acknowledged^56^.

**Table 1:** Mechanisms involved in the pathogenesis of viral-induced parkinsonism.

Mechanism	Type of neuronal damage
Virus tropism for basal ganglia, replication and subsequent neuronal lysis	Direct
Microglia activation and release of pro-inflammatory factors and T cell response	Indirect
Hypercytokinemia and loss of vascular integrity	Indirect
Hypoxic brain injury	Indirect

## SARS-CoV-2 receptors and cellular uptake

There is a wide diversity of proteins, particularly glycoproteins, that act as cellular receptors for coronavirus spike proteins^57^.

SARS-CoV-2 shares 70–80% of its genome with SARS-CoV and a smaller but significant homology with MERS-CoV^58^. This homology extends to the S protein^58^ that is the point of attachment to plasma membrane proteins which act as viral receptors for cellular infection. The S protein is thought to require a priming step in which it is cleaved by a cellular protease, which for SARS-CoV and SARS-CoV-2 is reported to be the cellular serine protease, TMPRSS2^59^.

The extensive research devoted to determining how the binding of the virus leads to cellular endocytosis of the virus, leading ultimately to RNA translation, transcription, and viral replication, will not be reviewed here.

At this time it appears that the main protein responsible for cellular accumulation of SARS-CoV-2 is angiotensin-converting enzyme 2 (ACE2)^27,60,61^, an enzyme that converts angiotensin II to angiotensin. ACE2 also acts as a receptor for several other coronavirus, including SARS-CoV^62,63^. The distribution of ACE2 throughout the body and brain is discussed below.

Recent in-silico studies^64,65^ propose that, in addition to ACE2, a second mechanism may enable cellular endocytosis of SARS-CoV-2. Similar to MERS-CoV, and in contrast to SARS-CoV, SARS-CoV-2 appears to display high binding affinity to sialic acid residues, providing an additional candidate for binding. Sialic acid residues are found on plasma membrane proteins of many cell types, including neurons, and are very highly expressed in the upper respiratory tract.

Additional observations that may test the predictions from in-silico reports implicating a role for sialic acid residues as SARS-CoV-2 receptors include (1) efficacy of the therapeutic use of lactoferrin^66^, an antiviral agent that interacts with sialic acid residues; (2) an ongoing clinical trial of DAS181 (https://clinicaltrials.gov/ct2/show/NCT04324489), a drug designed to block viral access by cleaving sialic acid; (3) that the shedding pattern of SARS-CoV-2 infection is different from that of SARS-CoV and more similar to that of “standard” influenza^66^, where sialic acid receptors play a major role; (4) a bioinformatic study reports binding of S-protein to sialic acid glycans in a region close to that identified by the in silico studies^65,67^. To our knowledge, while links between sialic acid and neurotropism for mouse hepatitis virus^68^ and adenoviruses^69^ have been suggested, there are no published investigations on this alternative pathway for SARS-CoV-2 interaction in the nervous system.

There are additional strong candidates for receptors for the virus, including the lectin CD209L (also known as L-SIGN), which acts as a receptor for the SARS virus^62,70^. This should be analyzed both in the nervous system and additional tissues, as well as other suggested candidate coronavirus receptors, most of which are highly charged and glycosylated^57^.

## Potential neurotropism of COVID-19 virus

At this time, we know very little about SARS-CoV-2 in the brain. Post-mortem studies on patients with SARS, however to have suggested the presence of viral particles in central nervous system (CNS) tissue^71,72^.

A recent publication examining the localization of SARS-CoV-2 in 27 people who died from COVID-19 demonstrated that 36% had apparently low levels of SARS-CoV-2 RNA and proteins in the brain, although they did not report the cellular localization or regions examined, and the signals may not have been present within the brain parenchyma^73^. A second study similarly reports detectable SARS-CoV-2 RNA in four of 12 brain samples, although again the signal may not have been from brain parenchymal cells^74^.

While there is, at this time, little evidence that SARS-CoV-2 enters the brain parenchyma, there are multiple means by which the virus might be able to do so^75^. Preclinical animal studies (reviewed by Natoli et al.^76^) report that after intranasal inoculation of SARS‐CoV in transgenic mice that overexpress human ACE2^77^, or MERS-CoV in mice overexpressing human dipeptidyl peptidase 4^78^, SARS‐CoV and MERS-CoV can invade the brain, possibly via transit through the olfactory nerves, to reach CNS nuclei, including thalamus and brainstem; we note, however, that these mice over-express the viral receptors, and these reports do not model normal infection routes.

Trans-synaptic transfer has been documented in rat and pig for other types of coronavirus, including hemagglutinating encephalomyelitis virus (HEV)^79–81^ and avian infectious bronchitis virus (IBV, also known as avian coronavirus)^82^, in both in vitro and in vivo studies.

Coronavirus might also reach the CNS via the hematogenous or lymphatic route, although this seems unlikely in early phases of the disease, as particles of SARS-CoV were not detected in non-neuronal cells in human post-mortem brain tissue^71,72^.

One potential mechanism for SARS-CoV-2 RNA presence within the CNS is blood-brain barrier (BBB) breakdown due to the cytokine storm associated with peripheral viral infection. It is well established that pro-inflammatory cytokines associated with inflammation and/or SARS-CoV-2 viral infection, such as tumor necrosis factor (TNF) and interleukin 1 beta (IL-1beta), mediate BBB breakdown^83^. This breakdown could either be long-term, similar to the one observed in neurodegenerative diseases allowing for infiltration of immune cells and viral particles, or transient, resulting in encephalitis^84,85^.

We note that while there are at this time several millions of SARS-CoV-2 infected individuals, there are only a few reports suggesting possible encephalitis, and only two that show evidence of COVID-19 virus in the CSF as assessed by reverse transcription polymerase chain reaction (RT-PCR). This suggests that even with the presence of high viral load in the blood stream and severe inflammation, COVID-19 virus is unlikely to exhibit direct neurotropism, but rather appears to cause inflammatory-mediated brain responses^86^.

## Presence of SARS-CoV-2 receptors in the brain

ACE2 was identified in 2000 as a novel carboxypeptidase that cleaves the vasoconstrictor angiotensin II to the vasodilator angiotensin (1–7), in addition to cleaving several other peptides^87^. ACE2 is a transmembrane protein, and can itself be cleaved near the transmembrane region and thereby be “shed” into a soluble form with anti-viral activity^88,89^, in part as the soluble form likely binds virus. Plasma membrane ACE2 is, confusingly, often referred to as the “ACE2 receptor”, but this is intended to convey that the protein, in addition to its normal function, can act as a receptor for virus—and not that it is a receptor for ACE2.

ACE2 is widely expressed in human tissue^90^ and appears to be increased by inflammatory signals including in macrophages^91^. Evidence supporting ACE2 expression in human brain parenchyma, however, remains poor, in contrast to clear expression in the brain vessels^92^. There is an extensive literature indicating that ACE2 may serve as a protective stress-induced response pathway^93–95^ and that its expression might be harnessed clinically for cardiac and neurological disorders, which will not be reviewed here.

In particular, while ACE2 expression has been demonstrated in CNS neurons in some animal models^96,97^, the presence of ACE2 in human CNS neurons is not well established, nor are specific brain regions or neuronal, astrocyte, microglial, immune or vascular cell types well characterized.

The ACE2 promoter harbors five hypoxia-responsive elements, and hypoxia may upregulate ACE2 via HIF1A-independent mechanisms^98^, but it has not yet been determined if hypoxia upregulates ACE2 in brain cells.

It is very important to compare the presence of brain ACE2, and perhaps of CD209L and molecules with sialic acid residues, in both control individuals and those with high inflammation. The expression of some of these “receptors”, including ACE2, can be enhanced by cytokines, such as interferon^99^, or other inflammatory responses^90^, and may be regulated by excitotoxicity^100^.

The Human Protein Atlas reports that ACE2 is not detected in normal human brain, but indicates low amounts in mouse brain (https://www.proteinatlas.org/ENSG00000130234-ACE2/tissue). As mentioned, an immunocytochemistry study of human brain tissue indicated that ACE2 is present in non-neuronal cells of vasculature in human brain tissue^92^, although that study did not define the precise cell types that express the receptor. A preprint of a single cell transcriptomic analysis suggests differential levels of ACE2 mRNA in different mouse brain regions^101^. Another preprint features a meta-analysis of single-cell and single-nucleus RNA sequencing datasets indicating co-expression of ACE2 and TMPRSS2 in oligodendrocytes^102^. However, additional studies are required to validate and localize protein co-expression in the CNS.

Because SARS-CoV-2 proteins can interact with host proteins involved in pathways that are altered during aging, including potential mitochondrial dysfunction, loss of proteostasis, autophagy dysfunction, inflammation, and endoplasmic reticulum stress, it is possible that SARS-CoV-2 infection may prompt protein misfolding and aggregation (Fig. 1)^103–105^. Of particular relevance for PD, recent studies have suggested that the aggregation-prone protein, alpha-synuclein, plays a role in the innate immune response to viral infections^106,107^.

It will be important to follow up and clinically monitor patients infected by COVID-19 virus, particularly those who developed specific neurological disturbances, such as sustained hyposmia^108^, syncope, and persistent confusion, given the relevance of these conditions to PD and PD dementia. Hyposmia is a well-recognized prodromal feature of PD^109^ as well as Alzheimer’s disease^110^ and may be due in part to dysfunction of inhibitory dopaminergic neurons in the olfactory bulb^111^. Although we do not yet know the precise mechanisms underlying hyposmia in COVID-19, it may be that patients who develop hyposmia become more susceptible to a neurodegenerative process or, alternatively, hyposmia may be a sign of peripheral inflammatory involvement of the olfactory mucosa. It is, therefore, reasonable to suggest specifically following up those COVID-19-linked cases where recovery is associated with sustained hyposmia after the acute illness of COVID-19 has subsided.

## COVID-19 and the possibility of a post-viral parkinsonism: clinical and molecular rationales

Some literature has already highlighted potential links between COVID-19 virus and neurodegenerative conditions, including suggestions regarding PD^104,112^. These are based on multiple observations:

1. The ability of coronaviruses to enter the CNS through the nasal cavity with subsequent neuronal death^77,78^, as shown in animal studies.
2. Hyposmia is well documented in COVID-19 patients without nasal obstruction and rhinorrhea^108,113,114^ and is also a common prodromal feature of PD^115^.
3. Basal ganglia lesions may occur in the context of a thromboembolic encephalopathy in COVID-19^116^.
4. The presence of higher levels of antibodies against other coronaviruses that cause the common cold in the CSF of PD patients compared to healthy controls suggests a possible involvement of viral infection in the pathogenesis of PD^47^.
5. There are reports that ACE2 may be expressed in various regions of the nervous system^93,117^, although as detailed above, further neuropathological investigation is required. Given the interferon activation of this protein, it will be important to examine subjects with CNS inflammation or encephalitis.
6. The recent reports of syncope with no abnormal rhythms on cardiac device interrogation hint at a potential role for neurally-mediated syncope^34^ vs. orthostasis, suggesting the importance of these investigations for PD patients who often suffer from dysautonomia^118^.
7. A single case report of a patient who developed myoclonus and an acute but spontaneously reversible hypokinetic rigid syndrome, with DaTscan showing reduction of dopamine transporter uptake in the putamen as well as hyposmia^119^.
8. The angiotensin system, which is implicated in COVID-19 pathogenesis, may be important in neuroinflammatory and neurodegenerative mechanisms observed in PD^120,121^.
9. SARS-CoV-2 proteins can interact with human proteins involved in biological mechanisms that drive dysfunction of protein homeostasis that may lead to protein misfolding and aggregation (Fig. 1)^103,104^.
10. The release of cytokines may activate resident immune cells in the CNS and/or lead to their infiltration from the periphery that result in brain cell damage. Such cells may include activated T cells and microglia that may kill neurons^122–124^, astrocytes, and vascular cell types. This may occur through the selection of cells that specifically recognize presented antigens from the infection or previous infections, or via a general activation of cytotoxic cells that recognize other antigens, including autoantigens, such as those derived from alpha-synuclein which are implicated in PD, Lewy Body dementias, multiple system atrophy, and multiple sclerosis^125,126^. High levels of pro-inflammatory cytokines, such as TNF and IL-1beta, are associated with increased risk of PD, while use of non-steroidal anti-inflammatory drugs (NSAIDs) and anti-TNF biologics reduce the risk^127^. Anti-TNF biologics are currently under investigation for COVID-19.

Beyond the significant observational literature discussed above that suggests a relationship between viral infection and PD^51^, a number of preclinical studies have directly addressed this issue. Jang et al. examined the potential for a neurotropic Type A influenza virus (A/Vietnam, 1203/04, H5N1, a.k.a. bird flu) to induce parkinsonian pathology in mice. They found that this strain of influenza virus directly infected neurons, with particular affinity for circuits involved in PD. Subsequent to recovery from this infection, the mice exhibited ataxia, tremor, and bradykinesia^128^ as well as a transient but significant loss of dopaminergic neuron phenotype, an early neuroinflammatory program, long-lasting microgliosis and an increase in alpha-synuclein expression^129^.

Another neurotropic virus, the mosquito-borne alphavirus, Western equine encephalitic virus (WEEV) also induces post-encephalitic parkinsonism. Like the influenza virus, WEEV induced activation of microglia and astrocytes, selective loss of dopaminergic neurons in the substantia nigra pars compacta (SNpc) and behavioral abnormalities consistent with PD in mouse models^130^. Importantly, the common denominator of these viruses is that they enter the CNS and directly infect cells.

As we do not yet know if the SARS-CoV-2 virus directly infects CNS neurons, it is important to determine if non-neurotropic viruses also have the potential to contribute to development of PD. The idea that a peripheral cytokine storm from non-neurotropic viruses can induce encephalitis has been suggested for many other viral infections, including the 1918 Spanish influenza (Type A H1N1)^131,132^ as well as respiratory syncytial virus^133^.

Notably, the pandemic 2009 H1N1 (CA/09) influenza virus does not infect neurons in the central, peripheral or enteric nervous systems, but can nevertheless induce a significant inflammatory response in the CNS, including within the SNpc. Evidence that an indirect neuroinflammatory mechanism of this sort might increase the risk of parkinsonism is that mice infected with the 2009 H1N1 virus, after complete resolution of peripheral infection, displayed a higher level of SNpc DA neuron death after injection with the parkinsonian neurotoxin, 1-methyl-4-phenyl-1,2,3,6-tetrahydropyridine (MPTP). Administration of an influenza vaccine or the neuraminidase inhibitor oseltamivir (Tamiflu) protected against the synergistic response to the neurotoxin^134^. In these preclinical studies, microgliosis and increase in inflammatory cytokines and chemokines in the brain were not due to invasion of CD4+/CD8+ T-cells from the periphery, suggesting that inflammatory cytokines released during peripheral infection passed through the blood-brain barrier^135^ and indirectly activated microglia, leading to a parkinsonian cascade.

Interestingly, influenza vaccination in humans enhanced levels of the anti-inflammatory cytokine interleukin 10 (IL-10)^136^, while prophylactic treatment with oseltamivir (Tamiflu) decreased disease severity of influenza in both human and mouse models, and did not appear to interfere with appropriate T cell responses to new influenza infection^137^. It may be that vaccination is protective for nervous system inflammatory responses even from viruses that do not infect neurons and astrocytes.

## The need for detailed autopsy studies

As deaths from SARS-CoV-2 infection continue, autopsy studies will play a key role in defining CNS pathology, including in patients with PD. However, due to increased precautions taken at the time of autopsy, relatively few brain autopsies are being performed. The U.S. Centers for Disease Control has issued guidance on autopsies for confirmed SARS-CoV-2 decedents and advises against performing procedures that generate aerosols, such as those used to remove the brain (https://www.cdc.gov/coronavirus/2019-ncov/hcp/guidance-postmortem-specimens.html#biosafety). Most studies thus far lack neuropathologic characterization altogether^138–142^, and an autopsy case series did not provide detailed neuropathologic descriptions^74^. Moreover, deaths occurring in nursing home and long-term care facilities, where a large subset of patients suffering from dementia reside, are less likely to result in autopsies. We thus expect a delay in understanding whether and how SARS-CoV-2 infection specifically alters neuropathology, including in PD.

Of the available studies with some neuropathologic data, one case series of ten autopsies documented no signs of encephalitis or CNS vasculitis, although the extent of neuroanatomic sampling was not provided^31^. A second study of COVID-19 autopsy findings included four brains that exhibited no encephalitis or neuronal necrosis, but mild hypoxemic changes in three of the four brains examined^143^. Although these studies have not identified specific neuropathologic alterations, the extent of involvement of the CNS in SARS-CoV-2 infection cannot be inferred from only 14 brains.

To establish how SARS-CoV-2 infection affects the CNS, the field will require detailed neuropathologic studies with thorough sampling of specific brain regions. At the Columbia University Medical Center, a current approach involves sampling of multiple neuroanatomic regions, including the cerebral cortex, watershed areas, white matter, olfactory system, hippocampus, amygdala, thalamus, hypothalamus, corpus striatum, pallidum, cerebellum, midbrain, pons, medulla oblongata, and cervical cord. We recommend that special attention be directed at documenting the presence and neuroanatomic distribution of hypoxia-related as well as inflammation-related pathologies, including leptomeningitis, encephalitis, and vasculitis.

## The clinical perspective

Clinical implications of SARS-CoV-2 infection on PD are largely speculative apart from two case series and case reports^45,46^. A community-based case control study in Italy of 12 PD COVID-19 cases suggested substantial worsening of motor and non-motor symptoms during mild to moderate COVID-19 illness, independent of age and disease duration^144^, in line with an original case report series by Antonini et al. In another survey across the Lombardy region of Italy, 105 probable COVID-19 cases were identified and the authors concluded that the risk, morbidity, and mortality in patients with mild-to moderate PD with COVID-19 did not differ from the general population^145^. Several viewpoints and editorials have been published on the topic in addition to extensive coverage in social media and journal viewpoint papers^146–151^.

Currently there is no robust evidence that having PD imparts an increased risk for susceptibility to COVID-19 or that COVID-19 confers a greater risk of PD, although, as noted above, there are reported cases of worsening of PD symptoms in infected patients, particularly in older frail patients on advanced therapies and one case report of development of an acute hypokinetic syndrome with hyposmia post COVID-19.

Broadly, the clinical impact of COVID-19 on PD could occur through multiple avenues:

1. Development of COVID-19-related symptoms, particularly high fever, severe respiratory distress, coagulopathy-related syndrome, fatigue, myalgias, and related impaired stress mechanisms.
2. Worsening of pre-existing dyspnea due to respiratory distress; dyspnea may exist in up to 39% of PD patients^152^.
3. In acutely ill patients admitted to hospital, confusion and delirium could occur (reported in over 25% of COVID-19 hospitalized subjects out of a survey of 3500 patients)^38^.
4. Worsening of specific symptoms, including motor symptoms as well as non-motor issues, such as pain, anxiety, sleep disturbances and fatigue, especially with reduced access to physical therapy and counseling^45,144^.
5. Social isolation and aggravation of underlying cognitive and behavioral symptoms, specifically anxiety^153^.
6. Possibility of post-traumatic stress disorder (PTSD) as observed in previous SARS and MERS pandemics^38^.
7. Increased levodopa requirement during acute admissions and need for non-oral dopaminergic therapies in some subjects with severe COVID-19 related symptoms^45^.
8. Potential for drug interaction of over the counter cough remedies with anti-parkinsonian drugs such as monoamine oxidase inhibitors.
9. Complexity in therapeutic management related to limitations of in-person consultations and admissions to hospital^147^.

The impact of severe infection (by default, implying a high viral load or a pro-inflammatory state) may lead to hospitalization and the need for supported breathing or mechanical ventilation, particularly in older PD patients with multimorbidity and a high frailty index^154^. The issue is further compounded because such patients may be on non-oral therapies (subcutaneous apomorphine, intrajejunal levodopa infusion, and deep brain stimulation (DBS)) for advanced PD^151^. Limited observations from admission of such cases around the world (personal communication) and the published case series suggest that such patients are particularly vulnerable, with high mortality rates and may have an increased levodopa requirement during the acute illness^45,46^. Pre-existing dyspnea of PD^152,155^, respiratory muscle bradykinesia^155^ in addition to a possible direct SARS-CoV-2-related brainstem-generated suppression of cough reflex and perhaps of autoregulation of blood flow may play additional negative roles^77,78,156,157^.

Fatigue has been commonly reported after many viral infections, most notably with Epstein-Barr virus^158^, and is evident in many non-PD cases with COVID-19^159^. Fatigue was also common in the series of PD cases reported^45^ and is an important contributor of quality of life^160^. Myalgia is also common after viral illnesses including COVID-19^40,161^, and in some cases of COVID-19 with PD, myalgia can be severe and involve muscles of the back. If these observations are confirmed in larger cohorts of PD patients with COVID-19, specific anti-fatigue/myalgia measures may need to be implemented^160^. Consideration for the use of amantadine-like drugs may be particularly relevant given their putative antiviral effects^162,163^; however specific clinical trials are lacking.

Social isolation and its impact on PD are a concern and has been called a “hidden sorrow” of the pandemic^164^. Social isolation may cause heightened anxiety, aggravation of pre-existing depression, the negative effects of stress on PD^165^, as well as lack of exercise. In the previous SARS and MERS epidemics, one in three hospitalized cases went on to develop a PTSD with 15% developing depression and anxiety at 1 year, and fatigue in more than 15%^38^. Anxiety in PD during COVID-19-related lockdown and consequent stress is widely reported during telephone consultations in many countries, and specific strategies for home care using telemedicine or remote counselling may need to be implemented.

**Fig. 2:**
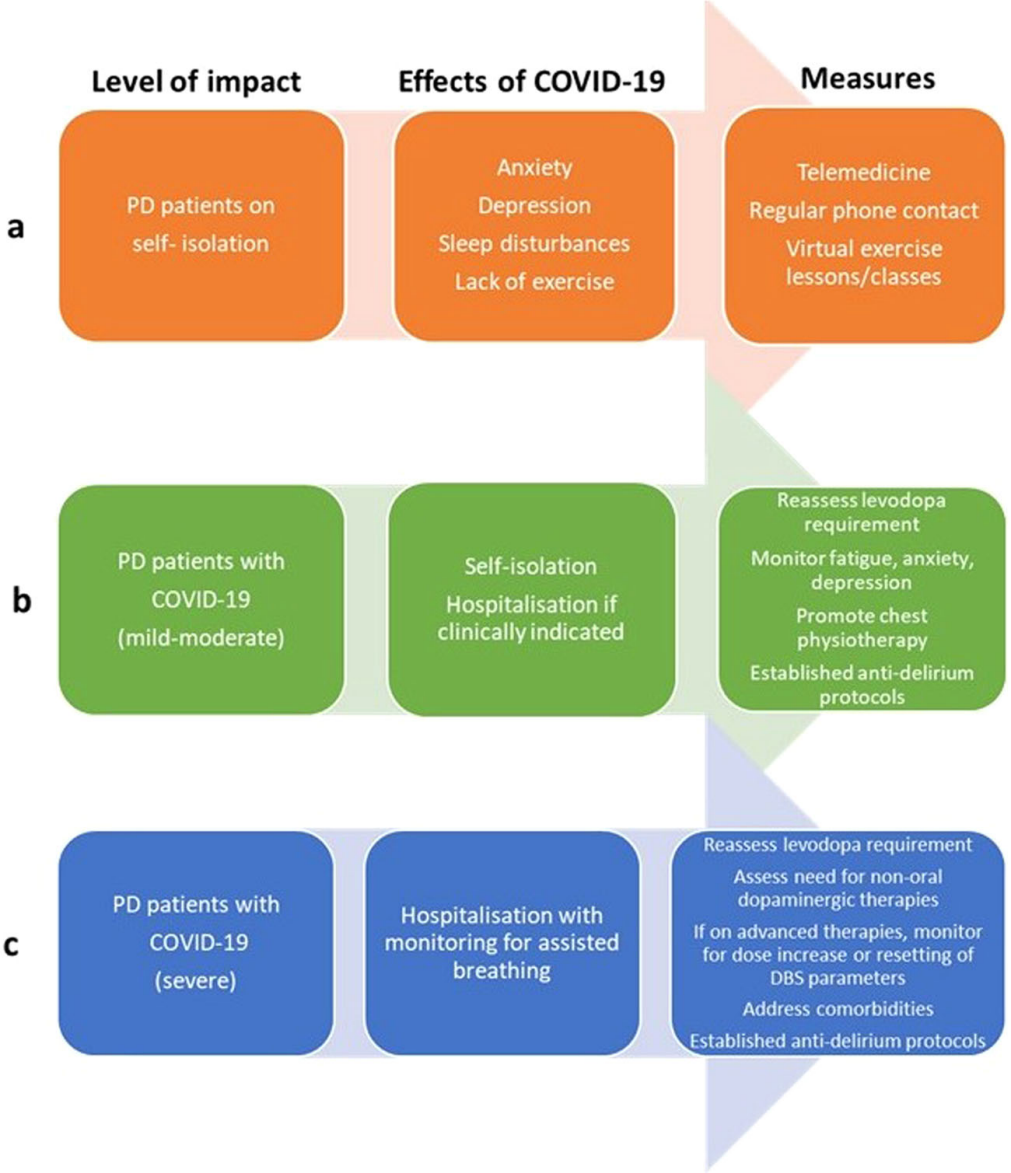
Flowchart identifying potential management issues in Parkinson’s disease patients. **a** Parkinson’s disease patients exposed to self-isolation or **b**, **c** infection with Coronavirus disease 2019 virus.

An overall consensus-led guideline for management of PD with varying grades of COVID-19 needs to be developed and circulated for implementation. A suggested template is provided in Fig. 2. These observations can be applied to the elderly as well as subjects with other neurodegenerative disorders, such as Alzheimer’s disease or amyotrophic lateral sclerosis.

## Conclusions

There has been a large number of papers on COVID-19 and PD speculating on etiology, risks and consequences, in addition to two documented case series of PD with COVID-19. We attempt to prove a critical approach to these observations from currently available clinical and molecular insights.

- The COVID-19 pandemic has led to an unprecedented crisis for older people globally. There is a broad range of COVID-19 symptoms, perhaps related to pre-existing conditions and in part to different modes of viral entry and the presence of T cells that are reactive to prior coronavirus infections. The neurological manifestations may be related to inflammation involving capillaries and the blood-brain barrier, hypoxemia, and thrombosis acting as triggers for seizures or leading to ischemic or hemorrhagic strokes.
- Neuropathology studies have not yet clearly answered the central issue of whether the virus enters central nervous system neurons, astrocytes or microglia.
- In brain vasculature, the cell types that express virus have not yet been identified.
- There is no clear evidence in human neurons or astrocytes for expression of the protein ACE2, which is thought to act as the major viral receptor that enables viral entry. Such expression may, however, be activated by inflammation, and thus comparison of healthy and infected brains will be important.
- There is a variety of alternative viral receptors for coronavirus, including sialic acid residues, that are insufficiently characterized and may provide entry into neurons and astrocytes.
- In contrast to the 1918 influenza pandemic and avian flu, reports of encephalopathy in COVID-19 have been slow to emerge, and there are so far no documented reports of an induction of parkinsonism apart from a single report. While a role for the virus in causing or exacerbating Parkinson’s disease appears unlikely at this time, the aggravation of specific motor and non-motor symptoms is reported.
- As the prevalence of PD rises sharply in the older age group, particularly in those over the age of 80 years, a personalized approach in the management of PD patients affected by COVID-19 based on clinical and basic science evidence is required. In addition, it will be important to monitor subjects after recovery, particularly for those with persisting hyposmia.

## Data Availability

Data sharing not applicable to this article as no data sets were generated or analyzed during the current study.
